# Eligibility for Adjuvant Cyclin-Dependent Kinase 4/6 Inhibitors in Endocrine Receptor-Positive and HER2-Negative Early Breast Cancer by Age and Type of Surgery

**DOI:** 10.3390/cancers16193317

**Published:** 2024-09-27

**Authors:** Gilles Houvenaeghel, Jean-Marc Classe, Marie-Pierre Chauvet, Pierre-Emmanuel Colombo, Eva Jouve, Fabien Reyal, Emile Daraï, Roman Rouzier, Christelle Faure-Virelizier, Pierre Gimbergues, Charles Coutant, Chafika Mazouni, Anne-Sophie Azuar, Marc Martino, Catherine Bouteille, Monique Cohen, Alexandre de Nonneville

**Affiliations:** 1 Aix-Marseille Univ, CNRS, INSERM, Institut Paoli-Calmettes, Department of Surgical Oncology, CRCM, 13009 Marseille, France; 2 Institut René Gauducheau, Site Hospitalier Nord, 44800 St. Herblain, France; jean-marc.classe@ico.unicancer.fr; 3 Centre Oscar Lambret, 3 rue Frédéric Combenal, 59000 Lille, France; mp-chauvet@o-lambret.fr; 4 Institut du Cancer de Montpellier, 31 Rue de la Croix Verte, 34090 Montpellier, France; pierre-emmanuel.colombo@icm.unicancer.fr; 5 IUCT Oncopole, 1 Av. Irène Joliot-Curie, 31100 Toulouse, France; jouve.eva@iuct-oncopole.fr; 6 Institut Curie, 26 rue d’Ulm, 75248 Paris Cedex 05, France; fabien.reyal@curie.fr; 7 Hôpital Tenon, AP-HP, 4 Rue de la Chine, 75020 Paris, France; emile.darai@tnn.aphp.fr; 8 Centre François Baclesse, 3 Av. du Général Harris, 14000 Caen, France; r.rouzier@baclesse.unicancer.fr; 9 Centre Léon Bérard, 28 rue Laennec, 69008 Lyon, France; christelle.faure@lyon.unicancer.fr; 10 Centre Jean Perrin, 58 rue Montalembert, 63000 Clermont Ferrand, France; pierre.gimbergues@cjp.fr; 11 Centre Georges François Leclerc, 1 rue du Professeur Marion, 21000 Dijon, France; ccoutant@cgfl.fr; 12 Institut Gustave Roussy, 114 Rue Edouard Vaillant, 94805 Villejuif, France; mazouni@igr.fr; 13 Hôpital de Grasse, Chemin de Clavary, 06130 Grasse, France; as-azuar@chgrasse.fr; 14 Hôpital Saint Joseph, 26 Bd de Louvain, 13008 Marseille, France; martinom@ipc.unicancer.fr; 15 Institut Paoli-Calmettes, Department of Surgical Oncology, CRCM, 13009 Marseille, France; bouteillec@ipc.unicancer.fr (C.B.); cohenm@ipc.unicancer.fr (M.C.); 16 Aix-Marseille Univ, CNRS, INSERM, Institut Paoli-Calmettes, Department of Medical Oncology, CRCM, 13009 Marseille, France

**Keywords:** abemaciclib, ribociclib, age, breast cancer, sentinel lymph nodes

## Abstract

**Simple Summary:**

Improved outcomes have been reported with adjuvant treatment combining cyclin-dependent kinase 4/6 inhibitors with endocrine therapy, in high-risk patients with ER-positive and HER2-negative breast cancer, regardless of age. In this real-world data analysis, MonarchE and NataLEE high-risk patients accounted for 9.5% and 33% of patients undergoing upfront surgery, respectively. Significantly higher eligibility rates were observed in patients who underwent a mastectomy, >70 years and ≤40 years for adjuvant abemaciclib and ribociclib, and in patients >80 years for ribociclib. A higher discontinuation rate for abemaciclib was reported in patients ≥65 years and it can be assumed that discontinuation rates may increase with more older patients. If the results of the NataLEE trial translate into clinical practice, the number of patients potentially eligible for adjuvant CDK4/6 inhibitors may increase, especially in the elderly population.

**Abstract:**

**Background:** Despite early diagnosis, approximately 20% of patients with ER-positive and HER2-negative breast cancer (BC) will experience disease recurrence. Improved survival has been reported with adjuvant treatment combining cyclin-dependent kinase 4/6 inhibitors with endocrine therapy, in high-risk patients with ER-positive and HER2-negative BC, regardless of age. Older patients have higher rates of ER-positive/HER2-negative BC than younger patients. **Methods:** In this real-world data analysis, MonarchE and NataLEE high-risk patients accounted for 9.5% and 33% of patients undergoing upfront surgery, respectively. Significantly higher eligibility rates were observed in patients who underwent a mastectomy, >70 years and ≤40 years for adjuvant abemaciclib and ribociclib, and in patients >80 years for ribociclib. **Results:** Eligibility rates in patients ≤40 years and >80 years who underwent mastectomy were 27.8% and 24.7% for abemaciclib, respectively, and 56.6% and 65.2% for ribociclib, respectively. A higher discontinuation rate for abemaciclib was reported in patients aged ≥65 years and it can be assumed that discontinuation rates may increase in even older patients. **Conclusions:** If the results of the NataLEE trial translate into clinical practice, the number of patients potentially eligible for adjuvant CDK4/6 inhibitors may increase, especially in the elderly population.

## 1. Introduction

Breast cancer (BC) is the most common cancer and the leading cause of cancer death in women. The incidence of BC increases with age and more than 30% of patients with newly diagnosed breast cancer are aged 65 years or older [1], and approximately 24.8% of patients are aged 70 years or older [1,2]. Between 1990 and 2023, the annual number of new cases of BC in women in France has doubled from 29,934 to 61,214 cases per year [3]. Half of this increase is due to population growth and aging. Age-specific trends show an average increase in BC of about +1% per year for all age groups, except for women in their 70s, for whom the increase is higher (+1.9%) [4].

The decline in BC mortality is the result of major therapeutic advances combined with an increase in the proportion of cancers diagnosed at an early stage, particularly through organized screening. However, this mortality benefit appears to be less evident in older patients and 60% of BC deaths occur in women aged 65 years and older [4].

Approximately 80% of BC have an ER-positive HER2-negative phenotype [2]. Elderly patients presenting with more advanced BC, partly due to the lack of systemic screening [5,6], have higher rates of ER-positive/HER2-negative phenotypes than younger cohorts, such as those included in pivotal studies [7], with higher rates of triple-negative (TN) BC and HER2-positive BC in young (≤40 years old) and very young (≤35 years old) patients [8]. Similarly, a recent study of 235,368 patients with early BC reported an increase in Luminal BC with increasing age: 46.9% in those aged <30 years; 87% in those aged 70–79 years; and 93.4% in patients aged 80 years or older [2].

In the large French cohort study reported by Dumas et al. [2], mastectomy rates for upfront surgery or after neoadjuvant chemotherapy were 41.2% in patients <40 years (4802/11,663), 24.8% in patients 70–79 years (9694/39,163), 43.4% in patients ≥ 80 years (8366/19,291), and 23.8% in patients 40–69 years (39,333/165,251). The mastectomy rate was 25.7% (39,362/153,379) in Luminal BC. Elderly BC patients are often undertreated compared to younger BC patients [9,10,11,12] and have higher rates of recurrence and mortality [10,13,14,15,16,17], with a 5-year survival rate of 82.4% in patients aged 70–79 years and 74% in patients over 80 years [13,17,18,19]. It should be noted that non-compliance with endocrine therapy and radiotherapy is higher in patients aged 80 years and over [16,20,21,22,23,24,25].

In addition, despite early diagnosis, around 20% of patients with ER-positive and HER2-negative BC experience disease recurrence within the first decade, and this persistent risk highlights the need for novel therapeutic strategies, particularly for those with high-risk disease [26]. The risk of distant recurrence was strongly correlated with initial TN status, with risks ranging from 10 to 41%, depending on TN status and tumor grade [27]. For patients with T1N0 BC, the risk of distant recurrence was 10% for grade 1 BC, 13% for grade 2, and 17% for grade 3, 20% for T1N1 with 1–3 nodes involved, 34% for T1N2 with 4–9 nodes involved, 19% for T2N0, 26% for T2N1 with 1–3 nodes involved, and 41% for T2N2 with 4–9 nodes involved. These probabilities are only slightly reduced by the risk of death from another cause, except for women aged over 70 or in poor health.

In high-risk patients, adjuvant abemaciclib in the MonarchE trial [28] and ribociclib in the NataLEE trial [29] showed significantly improved survival with a combination of cyclin-dependent kinase 4/6 inhibitors (CDK4/6i: ribociclib and abemaciclib) and endocrine therapy (ET) compared to patients receiving ET alone. The side effects of abemaciclib or ribociclib and ET are likely to be a significant limitation to compliance with these treatments over prolonged periods (3 years, 2 years, and 10 years, respectively), which could vary significantly depending on the age of the patients. In the MonarchE trial with adjuvant abemaciclib, most common adverse events (AEs) including diarrhea occurred early during treatment and were often low grade (diarrhea grade 1–2: 75.7% vs. 8.5%; grade ≥ 3: 7.8% vs. 0.2%, for abemaciclib + ET vs. ET alone, respectively). Management of these side effects led to 43% of patients requiring at least one dose reduction [28]. Dose reductions enabled treatment to be maintained in most cases, and the analyses did not reveal any negative impact on treatment efficacy [28]. Furthermore, during the treatment period, in both arms of the study, most patients reported being bothered “not at all” or “a little” by side effects [30]. More patients ≥65 years reported no diarrhea compared to younger patients (40% vs. 32%) at month 3 and the relative difference remained constant throughout the treatment period [30]. Overall and clinically relevant AE rates in patients treated with abemaciclib were similar between patients <65 years and older patients (49% vs. 54%), including grade 3 AEs [30]. However, dose reductions and treatment discontinuations due to AEs were more frequent in patients ≥65 years than in patients <65 years (dose reduction: 55% vs. 42%; treatment discontinuation: 38% vs. 15%) [30], and in each subgroup patients stopped treatment without prior dose adjustment (19% in patients ≥ 65 years versus 8% in patients <65 years) [30]. Finally, the long-term PRO data for MonarchE suggest that the toxicities of abemaciclib, including diarrhea, have no impact on patients’ overall quality of life when well managed. In the NataLEE trial, the initial dose of 400 mg was thought to improve the tolerability of ribociclib, but the safety profile was similar to conventional 600 mg dosing, and the discontinuation rate due to an adverse event was high (19% in the ribociclib arm vs. 4% in the placebo arm) [29].

Our study aimed to evaluate eligibility rates of adjuvant abemaciclib or ribociclib and ET among early BC patients who underwent upfront surgery, for all patients and according to age subgroups and breast conservative surgery (BCS; lumpectomy) or mastectomy.

## 2. Materials and Methods

From a large multicenter cohort (ClinicalTrials.gov ID NCT03461172), 17,097 ER-positive and HER2-negative early BC patients who underwent upfront surgery in 13 French centers between 1990 and 2023 were retrospectively selected. HR positivity was determined according to the French guidelines (estrogen and/or progesterone receptors by IHC with a 10% threshold for HR positivity).

The main recorded characteristics were age (≤40 years, 41–50, 51–74, and ≥75), tumor histology (ductal, lobular, mixt, or other), SBR grade 1 or 2, sentinel node (SN) status (pN0 or pN0(i+) or micrometastases or macrometastases), pT size (pT1, pT2 ≤ 30 mm, or pT2 > 30 mm), lymphovascular invasion (LVI), breast surgery (BCS or mastectomy), axillary surgery (sentinel lymph node biopsy (SLNB) or SLNB and completion axillary lymph node dissection (cALND) or ALND alone), Ki 67 (>20 or ≤20), adjuvant chemotherapy (AC), and post-mastectomy radiotherapy (PMRT). The R0/R1 status was not present in the data collection, but patients with conservative treatment and positive bank were all re-operated on, either by conservative retreatment or mastectomy (which was then the final procedure retained in the data)

Patients eligible for abemaciclib in the MonarchE trial [31] included pT3 and/or pT1–2 grade 3 with 1–3 pathological axillary lymph nodes, and pT1–2 grade 1–2 with ≥4 pathological axillary lymph nodes. In the NataLEE trial [32], the inclusion criteria were broader and included patients with stage II, N0, grade 2, and Ki 67 ≥ 20% or determined to be high-risk according to prognostic gene-expression signatures.

We determined eligibility for adjuvant abemaciclib and ribociclib for all patients and according to age <70 years or ≥70 years old, 70–74 years or 75–80 years or >80 years old, ≤40 years or 41–50 years old, and upfront BCS or mastectomy, in univariate analysis. Treatments received were analyzed in a regression analysis according to age <70 years or ≥70 years old, and 70–74 years or 75–80 years or >80 years old. Eligibility rates for adjuvant abemaciclib or ribociclib were then analyzed according to age, type of surgery, and adjuvant chemotherapy (AC) or not.

Statistics: Standard descriptive statistics were used to describe patient and tumor characteristics. All statistical tests were two-sided. The level of statistical significance was set at a *p*-value ≤ 0.05. Statistical analyses were performed using the SPSS 16.0 (SPSS Inc., Chicago, IL, USA).

## 3. Results

Population: Median age was 60 years (mean 59.9) for all patients: 40.2% (6338/17,097) ≥65 years. Characteristics of 17,097 ER-positive and HER2-negative early BC patients who underwent upfront surgery for all patients, for patients <70 years or ≥70 years old, 70–74 years or 75–80 years or >80 years old, ≤40 years or 41–50 years old, and for breast lumpectomy or mastectomy are shown in Table 1 and Table 2. All factors were significantly different between age subgroups, except tumor histology according to age 70–74 years or 75–80 years or >80 years.

Treatments administered according to age ≥70 years or <70 years old, and 70–74 or 75–80 or >80 years old, in regression analysis adjusted for pT size, SBR grade, histology, LN status, LVI, and type of breast surgery are shown in Table 3. Less AC, ALND, PMRT, and more mastectomies were observed in patients ≥70 years compared to <70 years and in patients >80 years compared to 70–74 years.

### 3.1. Eligibility for Adjuvant Abemaciclib or Ribociclib in All Patients

The overall eligibility rate for adjuvant abemaciclib was 9.46% (1617/17,097) and 32.91% (5627/17,097) for adjuvant ribociclib. The median age was 58 years (mean 58.3) in patients eligible for abemaciclib and 60 years (mean 60.1) in patients ineligible for abemaciclib, and 58.5 years (mean 58.9) and 61 years (mean 60.4) in patients eligible and ineligible for ribociclib, respectively. Patients were aged <65 years in 66.8% (1080/1617) of patients eligible for abemaciclib and 65.9% (3710/5627) of patients eligible for ribociclib. The number of patients eligible for adjuvant abemaciclib or ribociclib by pT size, pN status, and SBR grade is shown in Table 4. In addition, 17.0% of patients with pT3 BC were grade 3: 20.8% (97/466) of pT3pN1 with macrometastases, 12.5% (36/287) of pT3pN0 or pN0(i+), and 8.5% (5/59) of pT3pN1mi.

### 3.2. Eligibility for Adjuvant Abemaciclib or Ribociclib According to Age and Breast Surgery (Figure 1)

Eligibility rates according to age subgroups <70 years or ≥70 years, 70–74 years or 75–80 years or >80 years, ≤40 years or 41–50 years, and by BCS or mastectomy are shown in Table 5. Eligibility rates for abemaciclib and ribociclib were significantly higher in patients aged <70 years, higher in patients aged >80 years and 75–80 years compared to 70–74 years, higher in patients aged ≤40 years compared to 41–50 years, and higher for mastectomy compared to BCS.

We then determined the eligibility rates according to the combination of age and breast surgery (Table 5). Significant lower rates were observed for patients who underwent BCS, ≥70 years vs. >70 years, and 41–50 years vs. ≤40 years for adjuvant abemaciclib and ribociclib, and patients 70–74 years versus 75–80 and >80 years for ribociclib only. In patients who underwent a mastectomy, a significantly higher rate was only observed in patients >80 years compared to 70–74 years and 75–80 years.

Higher rates of adjuvant abemaciclib and ribociclib were reported in patients with AC versus without AC, and in patients who underwent mastectomy versus BCS among patients with AC and patients without AC.

Eligibility rates by combination of age and with or without adjuvant chemotherapy (Table 5). In patients without AC, higher rates were observed in patients ≥70 years versus <70 years, in elderly patients (>80 years versus 75–80 and 70–74 years) for abemaciclib and ribociclib, and in younger patients (≤40 years versus 41–50) for abemaciclib alone. Similar results were observed in patients with AC for patients ≥70 years versus <70 years, for elderly patients (>80 years versus 75–80 and 70–74 years) for abemaciclib, and only for patients ≥70 years versus <70 years for ribociclib. In younger patients with AC, lower rates were observed with ribociclib.

### 3.3. Regression Analysis for Adjuvant Abemaciclib and Ribociclib Adjusted for Age and BCS or Mastectomy for All Patients and according to Patients Treated with or without AC (Table 6)

In all subgroups analyzed, mastectomy versus BCS was significantly associated with higher eligibility for abemaciclib and ribociclib. Among patients ≥70 years, older patients (>80 years) were associated with higher eligibility for abemaciclib regardless of AC or no AC, and for ribociclib in patients without AC. For patients with AC that selected larger pT sizes, more pN1, and grade 3 BC, there was no significant association with age ≤40 years and 41–50 years with the eligibility rate for abemaciclib.

## 4. Discussion

The overall eligibility rate was 9.46% for adjuvant abemaciclib and 32.91% for adjuvant ribociclib. Higher rates were observed for patients <70 years old versus ≥70 years, but with higher rates for patients >80 years versus 70–74 and 75–80 years, and for patients ≤40 years versus >40 years, and significantly higher for mastectomy versus BCS.

High-risk patients: High-risk patients had distant recurrence rates of 20% for T1N1 with 1–3 nodes involved, 34% for T1N2 with 4–9 nodes involved, 19% for T2N0, 26% for T2N1 with 1–3 nodes involved, and 41% for T2N2 with 4–9 nodes involved [27]. Adjuvant abemaciclib in the MonarchE trial [31] and ribociclib in the NataLEE trial [29,32] showed significantly improved outcomes with the combination of cyclin-dependent kinase 4/6 inhibitors and ET compared to patients receiving ET alone. However, dose reductions and treatment discontinuations due to AEs were common with both treatments.

Eligibility: A previous retrospective US study using the MonarchE combined clinicopathological classification [33] reported a higher rate with 14% (557/4028) of patients considered MonarchE high risk. Similarly, a 2010–2015 SEER registry analysis [34] reported that 12% (28,619/238,222) of the patients were considered as archE high-risk. We found more pN1 (1–3 involved nodes) at 56.4% (912/1617) and less pN2 (≥involved nodes) at 43.6% compared to the MonarchE population with 40% and 60% of pN1 and pN2, respectively. The distribution for adjuvant ribociclib in our cohort was 12.5% of pN2 (705/5627), 78% of pN1 (4388/5627), and 9.5% of pN0 (534/5627) compared to 28% of patients classified as N0 based on baseline characteristics in the NataLEE trial. In the SEER registry analysis [34], this distribution differs with 47% of patients with N1 and 53% with N2–3 BC. In our cohort, patients eligible for abemaciclib were more likely to have pT3 BC (32.5%: 525/1617) than in the MonarchE trial (21.9%: 1217/5556), and 14.4% (812/5627) of patients eligible for ribociclib in our cohort had pT3 BC. In our cohort, 11.3% of BC were grade 3 (1937/17,097): 42.3% of patients were eligible for abemaciclib (819/1937) compared to 40.0% (2150/5370) in the MonarchE trial and 65.3% of patients were eligible for ribociclib (1264/1937) in our cohort. In addition, the mean age was higher in our cohort in patients eligible for abemaciclib (58 vs. 51 years in MonarchE) and in patients eligible for ribociclib (58.9 vs. 52 years in NataLEE). We found a higher proportion of patients aged ≥65 years eligible for abemaciclib (33.2%: 537/1617) than in the MonarchE trial (15.08%: 850/5637) and 34.1% (1917/5627) of patients eligible for ribociclib in our cohort were aged ≥65 years. Although the age of 65 years was chosen in the MonarchE trial, we analyzed the results both in patients considered old (≥70 years) and very old (>80 years), and in young or premenopausal (<50 years) and very young (≤40 years) patients.

Mastectomy and age: Higher mastectomy rates were reported in patients <40 years (41.2%) and in patients ≥80 years (43.4%) compared to the age subgroups 40–69 years (23.8%) and 70–79 years (24.8%) for upfront surgery or after neoadjuvant chemotherapy for all tumor subtypes [2]. Similarly, for ER-positive and HER2-negative BC, we observed higher mastectomy rates in older patients (39.4%, 23.7%, and 15.6% for patients aged >80 years, 75–80 years, and 70–74 years, respectively), and in younger patients (35.3% and 24.8% for patients aged ≤40 years and 41–50 years, respectively), with significant differences in multivariate analysis (Table 3). These rates may be due to patient preference and/or more advanced BC in older patients.

In addition, ER-positive and HER2-negative BC are more common in elderly patients [2,7]. As a result, a higher proportion of elderly patients may be eligible for adjuvant abemaciclib or ribociclib, and dose reductions and discontinuations due to AEs may increase in these patients. Tolaney et al. [30] reported a higher rate of abemaciclib discontinuation in patients aged ≥65 years and it can be assumed that discontinuation rates may increase with older patients. Unfortunately, the lower dose of ribociclib in the adjuvant setting (400 mg) did not show an improved tolerance profile compared with the metastatic setting (600 mg). This might represent a concern regarding observance and continuation in older patients with a high eligibility rate (30.6% and 43.5% in patients aged 75–80 years and >80 years, respectively) if ribociclib becomes available in clinical practice according to the NataLEE trial.

Prognostic gene-expression signatures are used to identify high-risk patients for the indication of AC but may not be sufficient to identify high-risk patients eligible for abemaciclib or ribociclib. Indeed, the NataLEE trial used a high genomic risk signature to assess eligibility for pT2 pN0 grade 2 breast cancer (BC) patients. However, only 17 patients met these criteria, resulting in a limited ability to draw meaningful conclusions from this aspect of the trial [29].

Our study has certain limitations. As a retrospective analysis, this study inherently suffers from selection bias and limitations in data availability. Moreover, the following limitations are also noted: the long time frame of inclusions with significant advancements having been made in treatment protocols; the study of age subgroups without specification of menopausal status; the lack of an onco-geriatric assessment; and the lack of gene-expression signatures that could have provided additional information on a minority population in the NataLEE trial. HER2-low status was not reported. While prognostic differences between HER2-low status and HER2 IHC 0 breast cancers have been reported, most studies found small or no differences in prognosis after adjusting for HR status [35,36,37], including in CDK4/6i-treated patients [38,39]. We also acknowledge the broader context of evolving breast cancer treatments and the future direction of personalized medicine [40].

## 5. Conclusions

Although ER-positive and HER2-negative BC is known to carry the better prognosis among BC subtypes, high-risk patients have a significantly increased risk of recurrence. This real-world data analysis, based on a large multicenter cohort, identifies up to 9.5% and 33% of high-risk patients according to MonarchE and NataLEE, respectively. Significantly higher eligibility rates were observed in patients who underwent a mastectomy, >70 years, ≤40 years for adjuvant abemaciclib and ribociclib, and in patients >80 years for ribociclib.

These clinical factors identified in the present work underline the need for physicians to be particularly aware of the case of elderly subjects, given the higher rate of CDK4/6i discontinuation reported in patients ≥65 years of age. Specific observational studies in older patients with detailed analysis of geriatric assessment, tolerability, and outcomes in these patients, especially those over 70 or 80 years, are warranted.

## Figures and Tables

**Figure 1:**
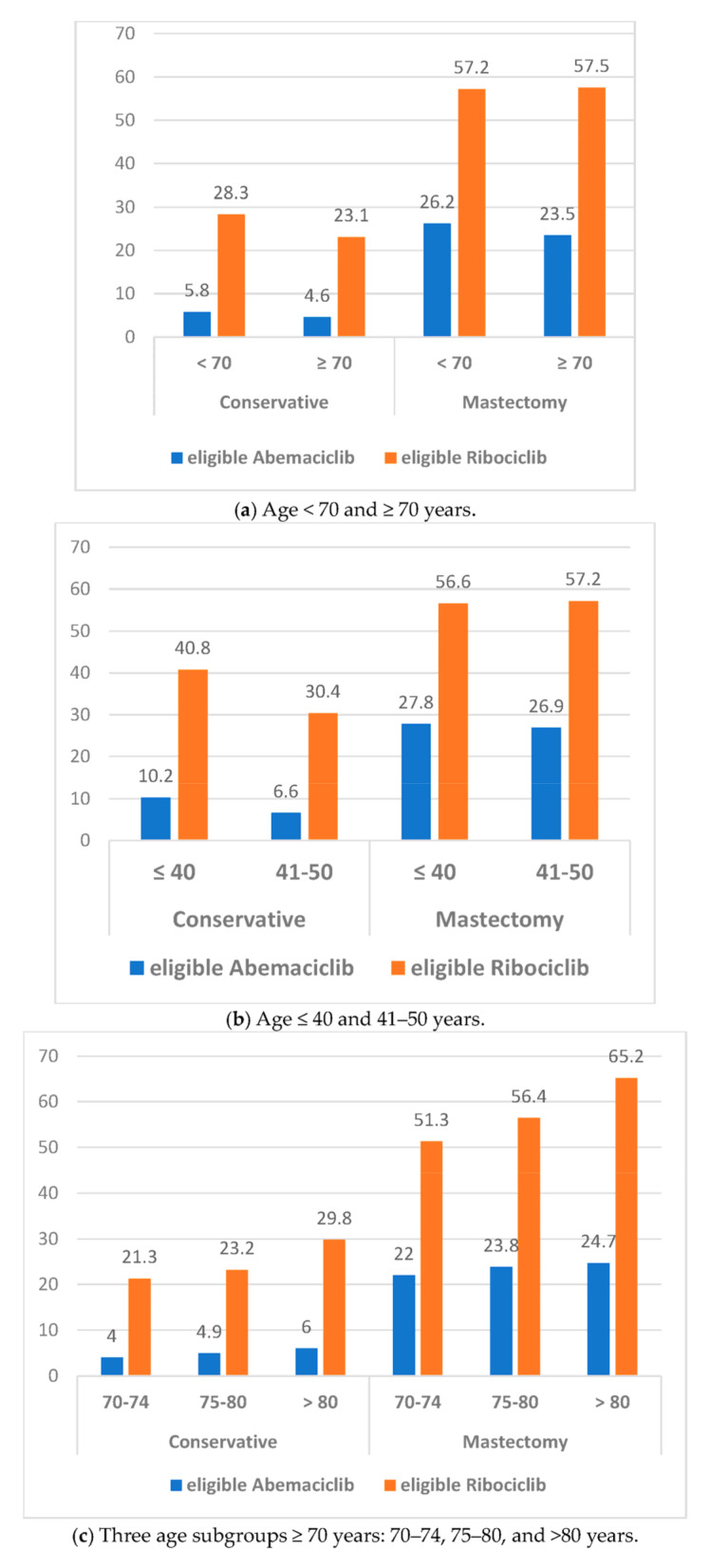
Eligibility rates for abemaciclib and ribociclib according to age subgroups and breast lumpectomy or mastectomy.

**Table 1:** Characteristics of 17,097 ER-positive and HER2-negative early BC patients who underwent upfront surgery for all patients, for patients <70 years or ≥70 years old, and breast lumpectomy or mastectomy.

		All Patients		<70 Years		≥70 Years			Conservative		Mastectomy		
		Nb	%	Nb	%	Nb	%	*p*	Nb	%	Nb	%	*p*
All patients		17,097		13,075	76.5	4022	23.5		13,385	79.8	3388	20.2	
Axillary surgery	SLNB	11,419	66.8	8479	64.8	2940	73.1	<0.0001	9632	72.0	1523	45.0	<0.0001
	SLNB + cALND	4086	23.9	3428	26.2	658	16.4		2910	21.7	1116	32.9	
	ALND	1592	9.3	1168	8.9	424	10.5		843	6.3	749	22.1	
Histology	Ductal	13,009	76.1	10,054	76.9	2955	73.5	<0.0001	10,527	78.6	2222	65.6	<0.0001
	Lobular	2821	16.5	2086	16.0	735	18.3		1887	14.1	901	26.6	
	Mixt	286	1.7	224	1.7	62	1.5		170	1.3	110	3.2	
	Others	981	5.7	711	5.4	270	6.7		801	6.0	155	4.6	
Grade	1	6519	38.1	5146	39.4	1373	34.1	<0.0001	5478	40.9	873	25.8	<0.0001
	2	8641	50.6	6423	49.1	2218	55.1		6568	49.1	1942	57.3	
	3	1937	11.3	1506	11.5	431	10.7		1339	10.0	573	16.9	
SN status (n = 15,704)	pN0sn	11,266	71.5	8421	69.8	2805	77.0	<0.0001	9475	74.6	1487	55.5	<0.0001
	pN0(i+)sn	477	3.0	390	3.2	87	2.4		350	2.8	118	4.4	
	pN1mi sn	1493	9.5	1226	10.2	267	7.3		1182	9.3	304	11.4	
	pN1 macro metastases sn	2508	16.0	2022	16.8	486	13.3		1695	13.3	769	28.7	
LN status	pN0	11,830	69.2	8874	67.9	2956	73.5	<0.0001	9869	73.7	1698	50.1	<0.0001
	pN0(i+)	461	2.7	378	2.9	83	2.1		344	2.6	111	3.3	
	pN1mi	1419	8.3	1162	8.9	257	6.4		1132	8.5	281	8.3	
	macrometastases	3387	19.8	2661	20.4	726	18.1		2040	15.2	1298	38.3	
	≥4 pN macro	705	4.1	525	4.0	180	4.5		265	2.0	434	12.8	
Breast surgery	Conservative	13,385	78.3	10,343	79.1	3042	75.6	<0.0001					
	Mastectomy	3388	19.8	2480	19.0	908	22.6						
	unknown	324	1.9	252	1.9	72	1.8						
pT size	pT1	12,114	70.9	9447	72.3	2667	66.3	<0.0001	10,426	77.9	1399	41.3	<0.0001
	pT2 < 30 mm	2703	15.8	1971	15.1	732	18.2		2026	15.1	646	19.1	
	pT2 ≥ 30 mm	1468	8.6	1055	8.1	413	10.3		779	5.8	685	20.2	
	pT3	812	4.7	602	4.6	210	5.2		154	1.2	658	19.4	
Age	≤40	835	4.9						530	4.0	295	8.7	<0.0001
	41–50	3478	20.3						2557	19.1	857	25.3	
	51–74	10,775	63.0						8913	66.6	1642	48.5	
	≥ 75	2009	11.8						1385	10.3	594	17.5	
LVI	No	12,890	75.4	9669	74.0	3221	80.1	<0.0001	10,325	77.1	2353	69.5	<0.0001
	Yes	2756	16.1	2209	16.9	547	13.6		1857	13.9	857	25.3	
	Unknown	1451	8.5	1197	9.2	254	6.3		1203	9.0	178	5.3	
Ki 67	>20	1211	18.1	895	19.0	316	16.0	<0.01	835	16.9	376	21.5	<0.001
	≤20	5469	81.9	3811	81.0	1658	84.0		4099	83.1	1370	78.5	
	Unknown	10,417		8369		2048			8451		1642		
Adjuvant chemotherapy	No	11,964	70.0	8487	64.9	3477	86.4	<0.0001	9965	74.4	1733	51.2	<0.0001
	Yes	5133	30.0	4588	35.1	545	13.6		3420	25.6	1655	48.8	
PMRT	No	1323	39.8	862	35.4	461	51.8	<0.0001					
	Yes	2002	60.2	1573	64.6	429	48.2						
	Abemaciclib eligibility	1617	9.5	1265	9.7	352	8.8	0.043	741	5.5	862	25.4	<0.0001
	Ribociclib eligibility	5627	32.9	4400	33.7	1227	30.5	<0.0001	3630	27.1	1941	57.3	<0.0001

Legend: Nb: number, SLNB: sentinel lymph node biopsy, cALND: completion axillary lymph node dissection, SN: sentinel node, LN: lymph node, LVI: lymphovascular invasion, and PMRT: post-mastectomy radiotherapy.

**Table 2:** Characteristics of 17,097 ER-positive and HER2-negative early BC patients who underwent upfront surgery according to age category.

		70–74 Years		75–80 Years		>80 Years			≤40 Years		41–50 Years		
		Nb	%	Nb	%	Nb	%	*p*	Nb	%	Nb	%	*p*
All patients		2013	50.0	1257	31.3	752	18.7		835	19.6	3421	80.4	
Axillary surgery	SLNB	1479	73.5	941	74.9	520	69.1	<0.0001	470	56.3	2121	62.0	0.003
	SLNB + cALND	358	17.8	199	15.8	101	13.4		263	31.5	984	28.8	
	ALND	176	8.7	117	9.3	131	17.4		102	12.2	316	9.2	
Histology	Ductal	1511	75.1	906	72.1	538	71.5	0.146	734	87.9	2653	77.6	<0.0001
	Lobular	354	17.6	237	18.9	144	19.1		58	6.9	501	14.6	
	Mixt	23	1.1	27	2.1	12	1.6		12	1.4	69	2.0	
	Others	125	6.2	87	6.9	58	7.7		31	3.7	198	5.8	
Grade	1	758	37.7	419	33.3	196	26.1	<0.0001	201	24.1	1303	38.1	<0.0001
	2	1067	53.0	704	56.0	447	59.4		447	53.5	1725	50.4	
	3	188	9.3	134	10.7	109	14.5		187	22.4	393	11.5	
SN status (n = 15,704)	pN0sn	1481	79.5	869	75.3	455	72.3	0.003	217	54.5	2098	66.9	<0.0001
	pN0(i+)sn	43	2.3	30	2.6	14	2.2		31	7.8	101	3.2	
	pN1mi sn	123	6.6	83	7.2	61	9.7		64	16.1	360	11.5	
	pN1 macrometastases sn	215	11.5	172	14.9	99	15.7		86	21.6	576	18.4	
LN status	pN0	1546	76.8	912	72.6	498	66.2	<0.0001	464	55.6	2208	64.5	<0.0001
	pN0(i+)	41	2.0	29	2.3	13	1.7		33	4.0	102	3.0	
	pN1mi	118	5.9	81	6.4	58	7.7		96	11.5	345	10.1	
	macrometastases	308	15.3	235	18.7	183	24.3		242	29.0	766	22.4	
	≥4 pN macro	71	3.5	61	4.8	48	6.4	<0.01	47	5.6	155	4.5	0.180
Breast surgery	Conservative	1657	82.3	935	74.4	450	59.8	<0.0001	530	63.5	2508	73.3	<0.0001
	Mastectomy	314	15.6	298	23.7	296	39.4		295	35.3	849	24.8	
	Unknown	42	2.1	24	1.9	6	0.8		10	1.2	64	1.9	
pT size	pT1	1492	74.1	834	66.3	341	45.3	<0.0001	517	61.9	2363	69.1	<0.0001
	pT2 < 30 mm	309	15.4	230	18.3	193	25.7		161	19.3	544	15.9	
	pT2 ≥ 30 mm	144	7.2	130	10.3	139	18.5		103	12.3	296	8.7	
	pT3	68	3.4	63	5.0	79	10.5		54	6.5	218	6.4	
LVI	No	1610	80.0	1011	80.4	600	79.8	<0.0001	520	62.3	2416	70.6	<0.0001
	Yes	243	12.1	174	13.8	130	17.3		265	31.7	716	20.9	
	Unknown	160	7.9	72	5.7	22	2.9		50	6.0	289	8.4	
Ki 67	>20	122	6.1	106	8.4	88	11.7	<0.0001	121	14.5	251	7.3	<0.0001
	≤20	751	37.3	514	40.9	393	52.3		210	25.1	1060	31.0	
	Unknown	1140	56.6	637	50.7	271	36.0		504	60.4	2110	61.7	

Legend: Nb: number, SLNB: sentinel lymph node biopsy, cALND: completion axillary lymph node dissection, SN: sentinel node, LN: lymph node, and LVI: lymphovascular invasion.

**Table 3:** Treatments administered according to age ≥70 years or <70 years old, and 70–74 or 75–80 or >80 years old, in regression analysis adjusted on pT size, SBR grade, histology, LN status, LVI, and type of breast surgery.

17,244 patients	≥70 vs. <70	OR	CI 95%	*p*
	Adjuvant chemotherapy	0.115	0.10–0.13	<0.0001
	Mastectomy vs. BCS	1.187	1.08–1.31	0.001
	ALND vs. SLNB	0.658	0.59–0.73	<0.0001
	PMRT vs. no PMRT	0.372	0.30–0.46	<0.0001
**4048 patients**	**70–74 vs. 75–80 and >80**	**OR**	**CI 95%**	***p***
Chemotherapy	75–80	0.411	0.32–0.53	<0.0001
	>80	0.04	0.025–0.065	<0.0001
Mastectomy vs. BCS	75–80	1.509	1.24–1.84	<0.0001
	>80	2.261	1.82–2.81	<0.0001
>ALND vs. SLNB	75–80	0.668	0.54–0.82	<0.0001
	>80	0.581	0.45–0.75	<0.0001
PMRT vs. no PMRT	75–80	0.901	0.60–1.35	0.613
	>80	0.66	0.43–1.00	0.051
**4256 patients**	**41–50 versus ≤ 40 years**	**OR**	**CI 95%**	***p***
	Adjuvant chemotherapy	0.460	0.37–0.57	<0.0001
	Mastectomy vs. BCS	0.618	0.52–0.74	<0.0001
	ALND vs. SLNB	1.059	0.85–1.32	0.605
	PMRT vs. no PMRT	1.113	0.77–1.62	0.577

Legend: OR: odd ratio, BCS: breast conservative surgery (lumpectomy), SLNB: sentinel lymph node biopsy, ALND: axillary lymph node dissection, PMRT: post-mastectomy radiotherapy, and vs: versus.

**Table 4:** Number of patients eligible for adjuvant abemaciclib or ribociclib according to pT size, pN status, SBR grade, and ≥4 pN+ rates.

Eligible for				Abemaciclib	Ribociclib	≥4 pN+		<4 pN+
				Nb	Nb	Nb	%	Nb
pT3 N1 macro	(all pT3 = 812)			466	466	220	47.2	246
pT3 N1mi (n = 59)	G3			5	5	0	0	5
	G1-2 (n = 54)			54	54	0	0	54
pT3 N0				287	287	0	0	287
pT2 N1 macro	G3			342	342	86	25.1	256
(n = 1474)	G1-2 (n = 1132)		≥4 pN+	246	246	246	21.7	886
			<4 pN+	886	886			
pT2 N1mi	G3			90	90	0	0	90
(n = 399)			≥4 pN+	0	0	0	0	
	G1-2 (n = 309)		<4 pN+	309	309	0	0	309
pT1 N1 macro	G3			188	188	24	12.8	164
(n = 1447)	G1-2 (n = 1259)		≥4 pN+	129	129	129	10.2	1130
			<4 pN+	1130	1130			
pT1 N1mi	G3			97	97	0	0	97
(n = 961)	G1-2 (n = 864)		<4 pN+	864	864	0	0	864
pT2 N0	G3			409	409	0	0	409
(n = 2298)	G1-2 (n = 1889)	Ki 67 > 20 (n = 125)	<4 pN+	125	125	0	0	125
		Ki 67 ≤ 20 (n = 872)	<4 pN+	872	872	0	0	872
		Ki 67 unknown (n = 892)	<4 pN+	892	892	0	0	892
pT1 N0			<4 pN+	9706	9706	0	0	9706
Total				17,097	17,097	705	4.3	16,392
Nb eligible				1617	5627			
% eligible				9.46	32.91			

Legend: Nb: number, G: grade. Numbers highlighted in grey correspond to patients eligible for abemaciclib. Numbers highlighted in blue correspond to patients eligible for ribociclib.

**Table 5:** Eligibility rates according to age subgroups <70 years or ≥70 years old, 70–74 years or 75–80 years or >80 years old, ≤40 years or 41–50 years old, and according to breast lumpectomy or mastectomy.

Eligibility Rate for			Abemaciclib			Ribociclib		
		All Patients	Nb	%	Chi2: *p*	Nb	%	Chi2: *p*
Age subgroups	<70 years	13,075	1265	9.7	0.043	4400	33.7	<0.0001
	≥70 years	4022	352	8.8		1227	30.5	
	70–74	2013	135	6.7	<0.0001	515	25.6	<0.0001
	75–80	1257	117	9.3		385	30.6	
	>80	752	100	13.3		327	43.5	
	≤40	835	139	16.6	<0.0001	388	46.5	<0.0001
	41–50	3421	397	11.6		1265	37.0	
Breast surgery	BCS	13,385	741	5.5	<0.0001	3630	27.1	<0.0001
	Mastectomy	3388	862	25.4		1941	57.3	
	Unknown	324	14	4.3		56	17.3	
BCS	<70 years	10,343	602	5.8	0.005	2926	28.3	<0.0001
	≥70 years	3042	139	4.6		704	23.1	
	70–74	1657	66	4.0	0.159	353	21.3	0.001
	75–80	935	46	4.9		217	23.2	
	>80	450	27	6.0		134	29.8	
	≤40	530	54	10.2	0.004	216	40.8	<0.0001
	41–50	2508	166	6.6		762	30.4	
Mastectomy	<70 years	2480	649	26.2	0.059	1419	57.2	0.460
	≥70 years	908	213	23.5		522	57.5	
	70–74	314	69	22.0	0.724	161	51.3	0.002
	75–80	298	71	23.8		168	56.4	
	>80	296	73	24.7		193	65.2	
	≤40	295	82	27.8	0.404	167	56.6	0.451
	41–50	849	228	26.9		486	57.2	
AC	No	11,964	287	2.4	<0.0001	1965	16.4	<0.0001
	Yes	5133	1330	25.9		3662	71.3	
No AC	<70 years	8487	108	1.3	<0.0001	1148	13.5	<0.0001
	≥70 years	3477	179	5.1		817	23.5	
	70–74	1656	37	2.2	<0.0001	253	15.3	<0.0001
	75–80	1098	60	5.5		263	24.0	
	>80	723	82	11.3		301	41.6	
	≤40	293	9	3.1	0.021	46	15.7	0.133
	41–50	1993	26	1.3		250	12.5	
AC	<70 years	4588	1157	25.2	0.001	3252	70.9	0.018
	≥70 years	545	173	31.7		410	75.2	
	70–74	357	98	27.5	<0.0001	262	73.4	0.130
	75–80	159	57	35.8		122	76.7	
	>80	29	18	62.1		26	89.7	
	≤40	542	130	24.0	0.198	342	63.1	0.001
	41–50	1428	371	26.0		1015	71.1	
No AC	BCS	9965	114	1.1	<0.0001	1318	13.2	<0.0001
	Mastectomy	1733	172	9.9		639	36.9	
AC	BCS	3420	627	18.3	<0.0001	2312	67.6	<0.0001
	Mastectomy	1655	690	41.7		1302	78.7	

Legend: Nb: number, BCS: breast conservative surgery (lumpectomy), and AC: adjuvant chemotherapy.

**Table 6:** Regression analysis for adjuvant abemaciclib and ribocilib adjusted on age and BCS or mastectomy for all patients and according to patients treated with or without AC.

		Abemaciclib				Ribociclib			
	Nb Patients	*p*	OR	CI 95%		*p*	OR	CI 95%	
				Inf	Sup			Inf	Sup
All patients	17,097								
BCS	13,385		1				1		
Mastectomy	3388	<0.0001	5.880	5.281	6.546	<0.0001	3.644	3.370	3.941
Unknown	324								
<70 years	13,075		1				1		
≥70 years	4022	0.001	0.811	0.713	0.923	<0.0001	0.811	0.749	0.878
≥70 years	4022								
BCS	3042		1				1		
Mastectomy	908	<0.0001	6.098	4.821	7.714	<0.0001	4.151	3.541	4.868
Unknown	72								
70–74 years	2013		1				1		
75–80 years	1257	0.222	1.183	0.904	1.548	0.118	1.140	0.967	1.343
>80 years	752	0.079	1.299	0.971	1.739	<0.0001	1.628	1.349	1.965
≤50 years	4256								
BCS	3038		1				1		
Mastectomy	1144	<0.0001	4.666	3.858	5.643	<0.0001	2.742	2.383	3.155
Unknown	74								
≤40 years	835		1				1		
41–50 years	3421	0.020	0.772	0.620	0.961	<0.0001	0.741	0.633	0.867
Without AC	11,964								
BCS	9965		1				1		
Mastectomy	1733	<0.0001	8.099	6.332	10.359	<0.0001	3.574	3.186	4.009
Unknown	266								
<70 years	8487		1				1		
≥70 years	3477	<0.0001	3.291	2.567	4.219	<0.0001	1.574	1.582	1.945
Without AC	3477								
BCS	2681		1				1		
Mastectomy	724	<0.0001	7.203	5.149	10.076	<0.0001	4.162	3.466	4.999
Unknown	72								
70–74 years	1656		1				1		
75–80 years	1098	0.001	2.011	1.311	3.085	<0.0001	1.543	1.263	1.886
>80 years	723	<0.0001	3.108	2.045	4.725	<0.0001	2.836	2.295	3.505
Without AC	2286								
BCS	1829		1				1		
Mastectomy	405	<0.0001	4.664	2.369	9.183	<0.0001	2.247	1.703	2.965
Unknown	52								
≤40 years	293		1				1		
41–50 years	1993	0.093	0.512	0.234	1.117	0.353	0.848	0.600	1.200
With AC	5133								
BCS	3420		1				1		
Mastectomy	1655	<0.0001	3.187	2.797	3.633	<0.0001	1.766	1.539	2.027
Unknown	58								
<70 years	4588		1				1		
≥70 years	545	0.001	1.384	1.134	1.690	0.033	1.252	1.019	1.538
With AC	545								
BCS	361		1				1		
Mastectomy	184	<0.0001	3.241	2.203	4.768	<0.0001	2.381	1.488	3.810
Unknown	0								
70–74 years	357		1				1		
75–80 years	159	0.194	1.317	0.869	1.995	0.681	1.098	0.704	1.712
>80 years	29	0.003	3.513	1.550	7.961	0.128	2.600	0.759	8.909
With AC	1970								
BCS	1209		1				1		
Mastectomy	739	<0.0001	3.255	2.635	4.021	<0.0001	1.826	1.485	2.246
Unknown	22								
≤40 years	542		1				1		
41–50 years	1428	0.196	1.170	0.922	1.484	<0.0001	1.473	1.193	1.819

Legend: Nb: number, OR: odd ratio, Inf: inferior, Sup: superior, BCS: breast conservative surgery (lumpectomy), and AC: adjuvant chemotherapy.

## Data Availability

Data supporting reported results can be found in the Paoli Calmettes Institute breast cancer database.
